# A revision of chromosome II (CD) mapping in *Chironomus plumosus* (Linnaeus, 1758) group (Diptera, Chironomidae)

**DOI:** 10.3897/CompCytogen.v6i3.2831

**Published:** 2012-08-09

**Authors:** Veronika V. Golygina, I. I. Kiknadze

**Affiliations:** 1 Institute of Cytology and Genetics SB RAS, Prosp. akademika Lavrentieva 10, Novosibirsk 630090, Russia; 2 Novosibirsk State University, ul. Pirogova, 2, Novosibirsk, 630090, Russia

**Keywords:** Chironomus plumosus group, karyotype, banding sequence, chromosome II, mapping

## Abstract

A revision of the main and alternative banding sequences in chromosome II (CD) has been made for all 14 species of the *Chironomus plumosus* (Linnaeus, 1758)group. A new version of mapping has been suggested for 10 out of 18 banding sequences of arm C and 12 out of 22 banding sequences of arm D. Mapping of 7 banding sequences has been done for the first time according to the Keyl-Devai system. Phylogenetic relationships of banding sequences of chromosome II have been discussed.

## Introduction

The *Chironomus plumosus* (Linnaeus, 1758) group of sibling species is a unique object for the study of patterns in linear reorganization of the genome during speciation, as it consists of species with mainly wide geographic ranges with natural populations at different stages of divergence (Butler et al. 1999; Gunderina et al. 1999a, b; Kiknadze et al. 2000; Golygina et al. 2007). What is more important, the possibility of mapping all karyotypes in the genus *Chironomus* Meigen, 1803against one standard species allows us to detect all chromosomal rearrangements that distinguish different species and reconstruct their phylogenetic relationship on the basis of karyological analysis. However, for conducting such a study it is very important to have high resolution photographic maps of karyotypes and a unified mapping system of polytene chromosomes. In our earlier work (Golygina and Kiknadze 2008) we extensively discussed all the difficulties facing a researcher who works with *Chironomus plumosus* group and presented a revision of mapping in chromosome I (AB). In this paper we present the results of revision of the main and alternative banding sequences in chromosome II (CD) for 14 *Chironomus plumosus* group species.

## Methods

Revision of chromosome II (CD) mapping was conducted for 14 *Chironomus plumosus* sibling species: *Chironomus agilis* Shobanov et Djomin, 1988, *Chironomus* sp. prope *agilis* (working name “*Chironomus agilis* 2”)(Kiknadze et al. 1991a), *Chironomus balatonicus* Devai, Wülker & Scholl, 1983, *Chironomus bonus* Shilova & Dzhvarsheishvili, 1974, *Chironomus borokensis* Kerkis, Filippova, Shobanov, Gunderina & Kiknadze, 1988, *Chironomus entis* Shobanov, 1989, *Chironomus muratensis* Ryser, Scholl & Wülker, 1983, *Chironomus nudiventris* Ryser, Scholl & Wülker, 1983, *Chironomus plumosus* (Linnaeus, 1758), *Chironomus sinicus* Kiknadze, Wang, Istomina & Gunderina, 2005, *Chironomus* sp. J (Kiknadze et al. 1991b), *Chironomus* sp. K (Golygina & Ueno, 2005), *Chironomus suwai* Golygina & Martin, 2003, *Chironomus usenicus* Loginova & Belyanina, 1994. High-resolution photomaps were created for mapping all the banding sequences in question.

Mapping of arms C and D was done according to the Keyl-Devai mapping system (Keyl 1962, Dévai et al. 1989). For banding sequences in arm C of *Chironomus balatonicus* the additional letter D was used for designation of regions 23 and 24, i.e. they are designated now as D23 and D24, as these regions originated in arm D and were transferred into arm C as a result of pericentric inversion.

Each banding sequence in each chromosomal arm is given a short designation as followes: three-letter abbreviation of the species name (for example, agi – for *Chironomus agilis*, bal – for *Chironomus balatonicus*, etc.) is followed by the name of the arm and the serial number of banding sequence in this arm (according to the order of its discovery), and prefixed by a letter that indicates its geographical distribution - p’ for Palearctic sequences, n’ for Nearctic sequences, or h’ for Holarctic sequences (e.g. p’balC1, n’entD4, h’pluD2 etc.).

Equipment in the Centre of Microscopical analysis of biological objects SB RAS in the Institute of Cytology and Genetics (Novosibirsk) was used for this work: microscope “Axioskop” 2 Plus, CCD-camera AxioCam HRc, software package AxioVision 4 (Zeiss, Germany).

## Results

### Arm C

**Figure 1.**
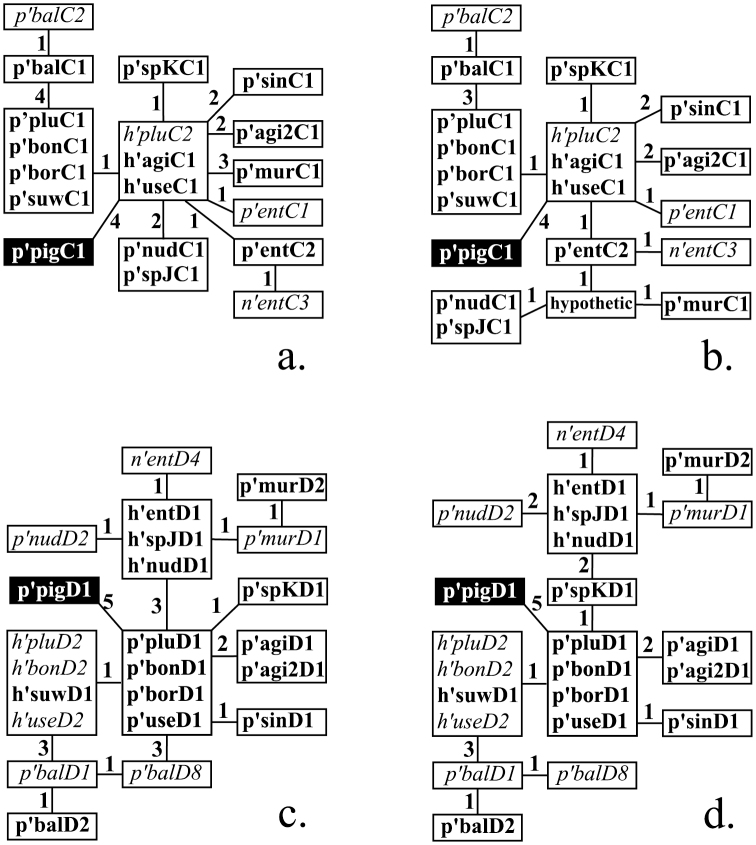
Phylogenetic relationship of main and alternative banding sequences in arms C and D before (**a**, **c**) and after (**b**, **d**) the revision. p’pluC1, h’entD1, n’entD4 etc. are the names of banding sequences considered in this study (please see ‘Methods’ for the rules of banding sequence designations). Main banding sequences are written in bold, alternative – in italic. Identical banding sequences enclosed in boxes, figures near the lines that connect boxes indicate numbers of inversion steps between banding sequences. The banding sequences p’pigC1 and p’pigD1 belong to *Chironomus piger* and are included into the dendrogramms as an outgroup.

Mapping of banding sequences of *Chironomus plumosus* sibling species according to Keyl-Devai system that was published by now is shown in Table 1. In total 18 banding sequences (14 main and 4 alternative) are considered in this study. A dendrogram of banding sequences constructed on the basis of published mapping is shown in Fig. 1a, where main banding sequences are written in bold and alternative banding sequences in italics. As can be seen, most of the banding sequences of different species were considered to be derivatives from h’pluC2 and its homologous banding sequences: eight blocks of sequences could be derived independently from h’pluC2 by one or more inversion steps.

**Table 1.** Mapping of arm C main and alternative banding sequences in *Chironomus plumosus* group before the revision. † – main banding sequences are marked by *, ‡ – papers with given version of the mapping are shown in parenthesis, § – mapping of this banding sequence is given with the same designations as in original paper, i.e. brackets indicate bands from arm D that were transferred into arm C as a result of pericentric inversion.

**Designation of banding sequence**	**Mapping of banding sequence**
h’agiC1*†	1a-2c 6c-f 7a-d 16a-17a 6hg 11d-15e 8a-11c 6b-2d 17b-22g C<br/> (Kiknadze et al. 1996b, 2004) ‡
p’agi2C1*	1a-2c 17a-16a 7d-a 6f-c 5a-6b 11c-8a 15e-11d 6gh 4i-2d 17b-22g C<br/> (Kiknadze et al. 2004)
p’balC1*	1a-2d 6c-e 7a-d 16a-17a 6h-f 11e-12d 4a-6b 11d-8a 15e-13a 3c-2e 17b-22g [24a-e 23a-c 23g-d 24fg] C§ (Kiknadze et al. 1996a)
p’balC2	not mapped in Keyl-Devai system
p’bonC1*	1a-2c 6c-f 7a-d 16a-17a 6hg 11d-12d 4a-6b 11c-8a 15e-13a 3c-2d 17b-22g C<br/> (Kiknadze et al. 2004)
p’borC1*	1a-2c 6c-f 7a-d 16a-17a 6hg 11d-12d 4a-6b 11c-8a 15e-13a 3c-2d 17b-22g C<br/> (Kiknadze et al. 2004)
p’entC1	1a 14a-11d 6gh 17a-16a 7a-d 6f-c 2c-1b 14b-15e 8a-11c 6b-2d 17b-22g C<br/> (Kiknadze et al. 2000)
p’entC2*	1a 11h-d 6gh 17a-16a 7a-d 6f-c 2c-1b 12a-15e 8a-11c 6b-2d 17b-22g C<br/> (Golygina 1999, Kiknadze et al. 2000, 2004)
n’entC3	1a 11h-d 6gh 17a-16a 7a-d 6f-c 2c 5a-6b 11c-8a 15e-12a 1b-2b 4i-2d 17b-22g C (Golygina 1999, Kiknadze et al. 2000)
p’murC1*	1a-2c 15e-a 8a-11c 6b-4a 6c-f 7a-d 16a-17a 6gh 11d-12d 14e-13a 3c-2d 17b-22g C (Kiknadze et al. 2004)
p’nudC1*	1a-2c 11d-15e 8a-11c 6b-2d 6c-f 7a-d 16a-17a 6gh 17b-22g C<br/> (Kiknadze et al. 2004)
p’pluC1*	1a-2c 6c-f 7a-d 16a-17a 6hg 11d-12d 4a-6b 11c-8a 15e-13a 3c-2d 17b-22g C<br/> (Butler et al. 1999, Golygina 1999, Golygina and Kiknadze 2001, Kiknadze et al. 2004)
h’pluC2	1a-2c 6c-f 7a-d 16a-17a 6hg 11d-15e 8a-11c 6b-2d 17b-22g C<br/> (Butler et al. 1999, Golygina 1999, Golygina and Kiknadze 2001)
p’sinC1*	1a-c 12d-11d 6gh 17a-16a 7d-a 6f-c 2c-1d 13a-15e 8a-11c 6b-2d 17b-22g C<br/> (Kiknadze et al. 2005)
p’spJC1*	not mapped in Keyl-Devai system
p’spKC1*	1a-2c 6c-f 7a-d 5c-6b 11c-8a 15e-11d 6gh 17a-16a 5b-2d 17b-22 C<br/> (Golygina and Ueno 2008)
h’suwC1*	1a-2c 6c-f 7a-d 16a-17a 6hg 11d-12d 4a-6b 11c-8a 15e-13a 3c-2d 17b-22g C<br/> (Golygina et al. 2003, Kiknadze et al. 2004)
p’useC1*	not mapped in Keyl-Devai system

According to our analysis, 11 banding sequences that belong to 8 species require a revision of mapping in this arm.

In our opinion, the most important changes should be made in mapping of banding sequences of *Chironomus* sp. prope *agilis*, *Chironomus balatonicus*, *Chironomus muratensis*, *Chironomus nudiventris*, and *Chironomus* sp. J, as we suggest a different way of their origination. Banding sequences of *Chironomus entis*, *Chironomus sinicus* and *Chironomus usenicus* required only minor corrections in the mapping of inversion breakpoints.

**Figure 2g–l.**
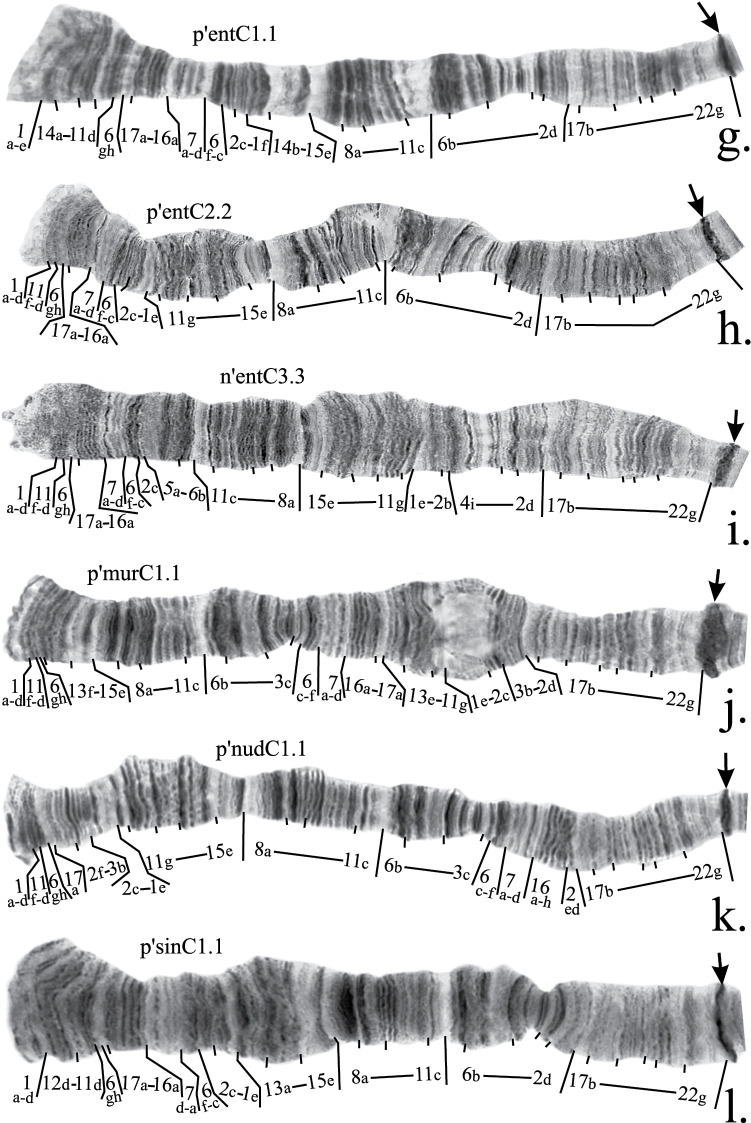
Mapping of banding sequences of *Chironomus plumosus* sibling species in arm C (*continued)*. **g** p’entC1.1 **h** p’entC2.2 **i** n’entC3.3 **j** p’murC1.1 **k** p’nudC1.1 **l** p’sinC1.1

The banding sequences of *Chironomus agilis*, *Chironomus bonus*, *Chironomus borokensis*, *Chironomus plumosus*, *Chironomus suwai* and *Chironomus* sp. K remain unchanged (Table 2, Fig. 2a, e, f).

**Table 2.** Mapping of arm C main and alternative banding sequences in *Chironomus plumosus* group after the revision. † – main banding sequences are marked by *, ‡ – parts of the sequences highlighted in bold indicate regions which mapping had been changed as a result of the revision, § – for banding sequences in arm C of *Chironomus balatonicus* additional letter D was used for designation of regions 23 and 24, i.e. they are designated now as D23 and D24, as these regions were initially originated in arm D and were transferred into arm C as a result of pericentric inversion. Moreover, regions that are affected by the pericentric inversion are given in the italic.

**Designation of banding sequence**	**Mapping of banding sequence**
h’agiC1*†	=h’pluC2
p’agi2C1*	**1a-e 5b-4h** 16h-a 7d-a 6f-c **2c-1f** **5c**-6b 11c-8a 15e-11d 6gh **17a** **4g**-2d 17b-22g C ‡
p’balC1*	1a-2c 6c-f 7a-d 16a-17a 6hg 11d-12d 4a-6b 11c-8a 15e-13a 3c-2d 17b-22g ***D24c-e D23ba D24b-D23c*** *D24fg C* §
p’balC2	**1a-2c 6c-f 7a-c 15e 8a-11c 6b-4a 12d-11d 6gh 17a-16a 7d 15d-13a 3c-2d 17b-22g *D24c-e D23ba D24b-D23c D24fg* C**
p’bonC1*	=p’pluC1
p’borC1*	=p’pluC1
p’entC1	**1a-e** 14a-11d 6gh 17a-16a 7a-d 6f-c 2c-**1f** 14b-15e 8a-11c 6b-2d 17b-22g C
p’entC2*	**1a-d 11f**-d 6gh 17a-16a 7a-d 6f-c 2c-**1e** **11g**-15e 8a-11c 6b-2d 17b-22g C
n’entC3	**1a-d** **11f**-d 6gh 17a-16a 7a-d 6f-c 2c 5a-6b 11c-8a 15e-**11g** **1e**-2b 4i-2d 17b-22g C
p’murC1*	**1a-d 11f-d 6gh 13f-15e** 8a-11c 6b-3c 6c-f 7a-d 16a-17a **13e-11g 1e-2c 3b**-2d 17b-22g C
p’nudC1*	**1a-d 11f-d 6gh 17a 2f-3b 2c-1e 11g**-15e 8a-11c 6b-**3c** 6c-f 7a-d 16a-h **2ed** 17b-22g C
p’pluC1*	1a-2c 6c-f 7a-d 16a-17a 6hg 11d-12d 4a-6b 11c-8a 15e-13a 3c-2d 17b-22g C
h’pluC2	1a-2c 6c-f 7a-d 16a-17a 6hg 11d-15e 8a-11c 6b-2d 17b-22g C
p’sinC1*	1a-**d** 12d-11d 6gh 17a-16a 7d-a 6f-c 2c-**1e** 13a-15e 8a-11c 6b-2d 17b-22g C
p’spJC1*	=p’nudC1
p’spKC1*	1a-2c 6c-f 7a-c 5c-6b 11c-8a 15e-11d 6gh 17a-16a 7d 5b-2d 17b-22g C
h’suwC1*	=p’pluC1
p’useC1*	**1a-2c 6c-f 7a-d 16a-17a 6hg 11d-15e 8a-11c 6b-2d 17b-22g C**

**Figure:**
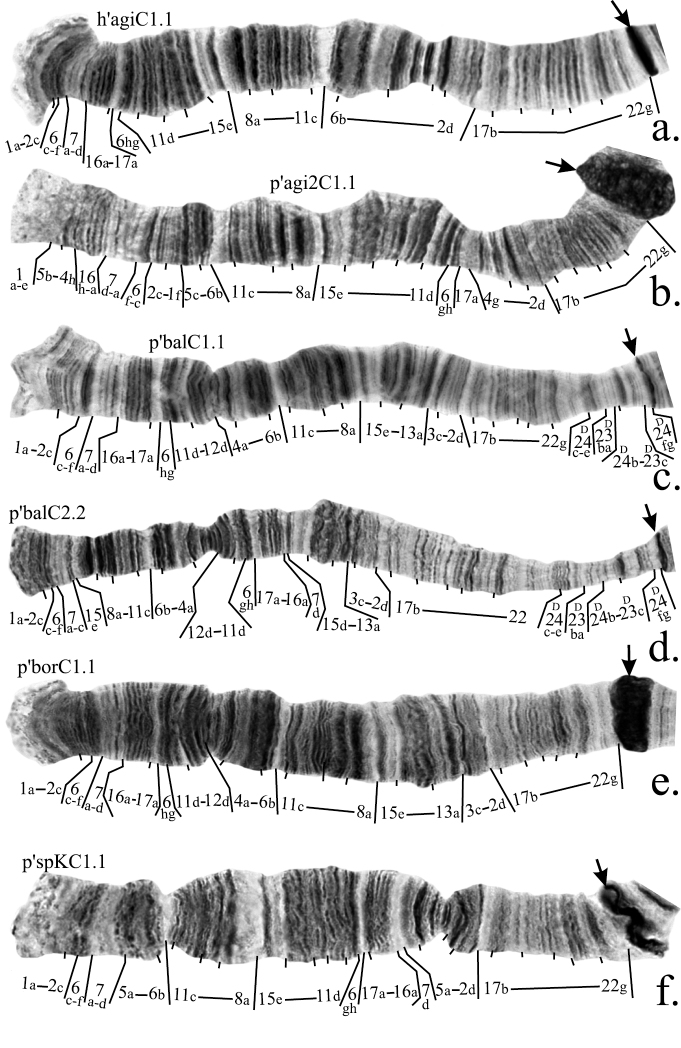
**Figure 2a–f**. Mapping of banding sequences of *Chironomus plumosus* sibling species in arm C. **a** h’agiC1.1 **b **p’agi2C1.1 **c** p’balC1.1 **d** p’balC2.2 **e** p’borC1.1 **f** p’spKC1.1. For banding sequences p’balC1.1 and p’balC2.2 letter D was used for designation of regions 23 and 24, i.e. they are designated now as D23 and D24, as these regions were initially originated in arm D and were transferred into arm C as a result of pericentric inversion. Centromeric bands designated by arrows.

**1. Revision of arm C mapping of *Chironomus* sp. prope *agilis* (*Chironomus agilis* 2)**

**Figure 3a–e.**
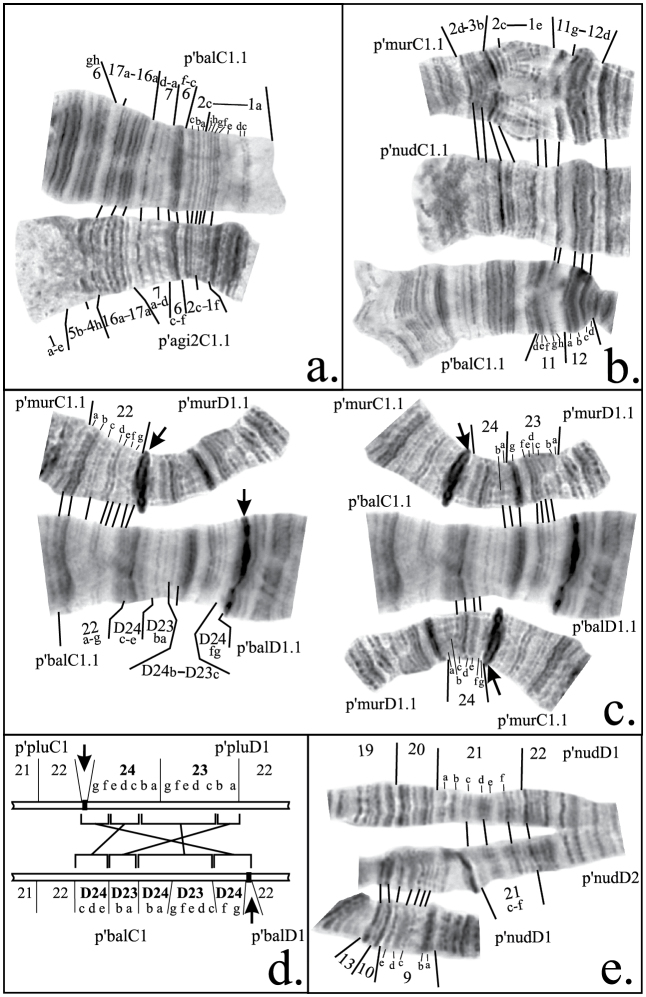
Mapping of some inversion breakpoints in species of *Chironomus plumosus* group in arms C and D. **a** comparison of parts of banding sequences p’balC1 and p’agi2C1 **b** comparison of parts of banding sequences p’murC1, p’nudC1 and p’balC1 **c** comparison of parts of banding sequence p’balD1 with p’murD1 and p’murC1 **d** schematic comparison of centromeric regions of chromosome CD of *Chironomus plumosus* and *Chironomus balatonicus* showing the structure of pericentric inversion in *Chironomus balatonicus*, brackets connected by lines indicate groups of bands affected by the inversion**e** comparison of parts of banding sequences p’nudD1 and p’nudD2. Abbreviations are as in Figure 2.

According to the previous mapping agi2C1 differs from agiC1 by two non-overlapping paracentric inversions (Kiknadze et al. 1991a). However, our analysis had shown that these two inversions are, in fact, overlapping and slightly bigger than was presumed previously. Due to these rearrangements the region 5b-4h- was transferred to the distal part of the arm, very close to the telomere, whereas bands 2c-1f could be found between regions 6f-c and 5c-f (Fig. 2b, 3a, Table 2).

**2. Revision of arm C mapping of *Chironomus balatonicus***

*Chironomus balatonicus* differs from all other species of *Chironomus plumosus* group by the presence of a complex pericentric inversion in chromosome CD. It was presumed previously (Kiknadze et al. 1996a) that the banding sequence in the centromeric region of *Chironomus balatonicus* was formed by four inversions (Table 1). However, comparison of p’balC1 with p’agiC1 and p’nudC1, which have the most clear banding structure in the centromeric region, allowed us to conclude that banding sequence in the centromeric regions of *Chironomus balatonicus* differs from other species by three inversions (Fig. 2c, 3 c, d, Table 2).

The banding sequence p’balC2 was previously mapped according to Maximova’s system only. It originated from p’balC1 by one simple inversion and its up to date mapping in the Keyl-Devai system is shown in Fig. 2d.

**3. Revision of arm C mapping of *Chironomus entis*, *Chironomus muratensis*, *Chironomus nudiventris*, and *Chironomus* sp. J**

It was presumed earlier that the main banding sequences of *Chironomus entis*, *Chironomus muratensis*, *Chironomus nudiventris* originated from h’pluC2 independently, and that p’spJC1 of *Chironomus* sp. J is identical to p’nudC1 (Fig. 1a). However, our analysis had shown that whereas this conclusion is true for p’entC1 and p’entC2, main banding sequences of *Chironomus muratensis* and *Chironomus nudiventris* (and, therefore, *Chironomus* sp. J, where the main banding sequence is indeed identical to p’nudC1)originated from p’entC2 through the same hypothetical banding sequence that at present does not occur in the banding sequence pools of these species (Fig. 1 b). Moreover, as the chromosome banding structure of *Chironomus muratensis* and *Chironomus nudiventris* is better than of *Chironomus entis*, comparison of their banding sequences with h’pluC2 also allowed us to correct mapping of breakpoints of p’entC2.

*Chironomus entis* has three banding sequences that have been found in the homozygous state and, therefore, are considered in this study: p’entC1, p’entC2 and n’entC3. Banding sequence p’entC1 differ from h’pluC2 by a simple inversion. A correction in the mapping of p’entC1 should be made for the left breakpoint of the inversion (Fig. 2g, Table 2).

As was mentioned above, the mapping of banding sequence p’entC2 is crucial for the mapping of n’entC3 and all banding sequences of *Chironomus muratensis*, *Chironomus nudiventris* and *Chironomus* sp. J. It differs from h’pluC2 by a simple inversion in the distal part of the arm with its left breakpoint located very close to the telomere. Analysis of these regions in the banding sequences of *Chironomus muratensis*, *Chironomus nudiventris* and *Chironomus* sp. Jallowed us to conclude that the real breakpoints of the inversion that distinguish p’entC2 from h’pluC2 fall between bands 1d and 1e on the left border, and 11f and 11h on the right border of the inversion (Fig. 2h, 3b, Table 2).

The mapping of n’entC3 should be corrected in accordance with mapping of p’entC1 (Fig. 2i, Table 2).

Thorough analysis of the main banding sequences of *Chironomus muratensis* and *Chironomus nudiventris* allowed us to conclude that they originated from p’entC2 through the hypothetical banding sequence:

1a-d 11f-d 6gh 17a-16a 7a-d 6f-c 2c 3c-6b 11c-8a 15e-11g 1e-2b 3b-2d 17b-22g C

Underline indicates simple inversion that distinguishes this banding sequence from p’entC2.

Both p’murC1 and p’nudC1 differ from this hypothetical banding sequence by simple inversions. Their revised mapping is shown in Table 2 and on Fig. 2j, k.

**4. Revision of arm C mapping of *Chironomus sinicus* and *Chironomus usenicus***

Mapping of p’sinC1 require only a minor revision. According to the previous version of the mapping, region 1 was divided by an inversion between bands 1c and 1d. However, we had not been able to locate band 1d near band 13a so we suggest that the left breakpoint of this inversion situated between band 1d and 1e (Fig. 2l, Table 2).

Until now the banding sequence h’useC1 has been mapped only partially (Loginova and Belyanina 1994), although it was indicated that it is identical to h’pluC2. We concur with this statement. Complete mapping of the h’useC1 in Keyl-Devai system is shown in Table 2.

Phylogenetic relationships of revised banding sequences in arm C of the *Chironomus plumosus* group species are shown in Fig. 1b.

### Arm D

Mapping for banding sequences in this arm that has been published so far is shown in Table 3. Phylogenetic relationship of banding sequences based on this mapping is shown in Fig. 1c. In total 22 banding sequences (14 main and 8 alternative) are considered in this study. Analysis of main and alternative banding sequences in this arm has shown that only minor changes in the mapping of inversion breakpoints are required for some banding sequences. The corrections in mapping should be made for nine banding sequences, belonging to *Chironomus agilis*, *Chironomus* sp. prope *agilis*, *Chironomus balatonicus*, *Chironomus entis*, *Chironomus muratensis*, *Chironomus nudiventris* and *Chironomus* sp. J. In addition, two banding sequences belonging to *Chironomus muratensis* and *Chironomus nudiventris* were mapped in Keyl-Devai system for the first time.

**Table 3.** Mapping of arm D main and alternative banding sequences in *Chironomus plumosus* group before the revision. † – main banding sequences are marked by *, ‡ – papers with given version of the mapping are shown in parenthesis.

**Designation of banding sequence**	**Mapping of banding sequence**
p’agiD1*†	1a-d 4a-7g 18a-d 8a-10a 13a-11a 3g-1e 10e-b 13b-14f 20b-18e 17f-14g 20c-24g C(Kiknadze et al. 2004) ‡
p’agi2D1*	1a-d 4a-7g 18a-d 8a-10a 13a-11a 3g-1e 10e-b 13b-14f 20b-18e 17f-14g 20c-24g C (Kiknadze et al. 2004)
p’balD1	1a-3g 10b-e 4a-7g 18a-d 8a-10a 13a-11a 13b-17f 18e-22e C<br/> (Kiknadze et al. 1996a)
p’balD2*	1a-3g 10b-e 4a-7g 18a-d 8a-9e 15e-13b 11a-13a 10a 16a-17f 18e-22e C<br/> (Golygina et al. 1996)
p’balD8	1a-3g 11a-13a 10a-8a 18d-a 7g-4a 10e-b 13b-17f 18e-22e C<br/> (Golygina et al. 1996)
p’bonD1*	1a-3g 11a-13a 10a-8a 18d-a 7g-4a 10e-b 13b-17f 18e-24g C<br/> (Kiknadze et al. 2004)
p’borD1*	1a-3g 11a-13a 10a-8a 18d-a 7g-4a 10e-b 13b-17f 18e-24g C<br/> (Kiknadze et al. 2004)
h’borD2	=h’pluD2 (Kerkis et al. 1988), not mapped according to Keyl-Devai system
h’entD1*	1a-2d 15e-16c 18d 8a-10a 13a-12a 18c-a 7g-4a 10e-b 13b-15d 2e-3g 11a-c 16d-17f 18e-24g C (Golygina 1999; Kiknadze et al. 2000)
n’entD4	1a-2d 15e-16c 18d 8a-d 19h-18e 17f-16d 11c-a 3g-2e 15d-13b 10b-e 4a-7g 18a-c 12a-13a 10a-9a 20a-24g C (Golygina 1999)
p’murD1	1a-i 11c-a 3g-2e 15d-13b 10b-e 4a-7g 18a-c 12a-13a 10a-8a 18d 16c-15e 2d-a 16d-17f 18e-24g C(Kiknadze et al. 2004)
p’murD2*	not mapped according to Keyl-Devai system
h’nudD1*	1a-2d 15e-16c 18d 8a-10a 13a-12a 18c-a 7g-4a 10e-b 13b-15d 2e-3g 11a-c 16d-17f 18e-24g C (Kiknadze et al. 2004)
p’nudD2	not mapped according to Keyl-Devai system
p’pluD1*	1a-3g 11a-13a 10a-8a 18d-a 7g-4a 10e-b 13b-17f 18e-24g C (Butler et al. 1999, Golygina 1999, Golygina and Kiknadze 2001, Kiknadze et al. 2004)
h’pluD2	1a-3g 10b-e 4a-7g 18a-d 8a-10a 13a-11a 13b-17f 18e-24g C<br/> (Butler et al. 1999, Golygina 1999, Golygina and Kiknadze 2001)
p’sinD1*	1a-2g 13a 10a-8a 18d-a 7g-4a 10e-b 13b-14h 3g-2h 12d-11a 15a-17f 18e-24g C (Kiknadze et al. 2005)
h’spJD1*	not mapped according to Keyl-Devai system
p’spKD1*	1a-3g 11a-13a 10a-8a 16d-13b 10b-e 4a-7g 18a-d 16e-17f 18e-24 C<br/> (Golygina and Ueno 2008)
h’suwD1*	1a-3g 10b-e 4a-7g 18a-d 8a-10a 13a-11a 13b-17f 18e-24g C<br/> (Golygina et al. 2003, Kiknadze et al. 2004)
p’useD1*	1a-3g 11a-13part 9a-e 18part 8d-4a 10ba 13part-17f 18part-24g C<br/> (Loginova and Beljanina 1994)
h’useD2	=h’pluD2 (Loginova and Beljanina 1994), not mapped according to Keyl-Devai system

Mapping of banding sequences of *Chironomus bonus*, *Chironomus borokensis*, *Chironomus plumosus*, *Chironomus sinicus*, *Chironomus* sp. K, *Chironomus suwai*, and *Chironomus usenicus* remains unchanged (Fig. 4e, f, l, m, Table 4).

**Figure 4a–f.**
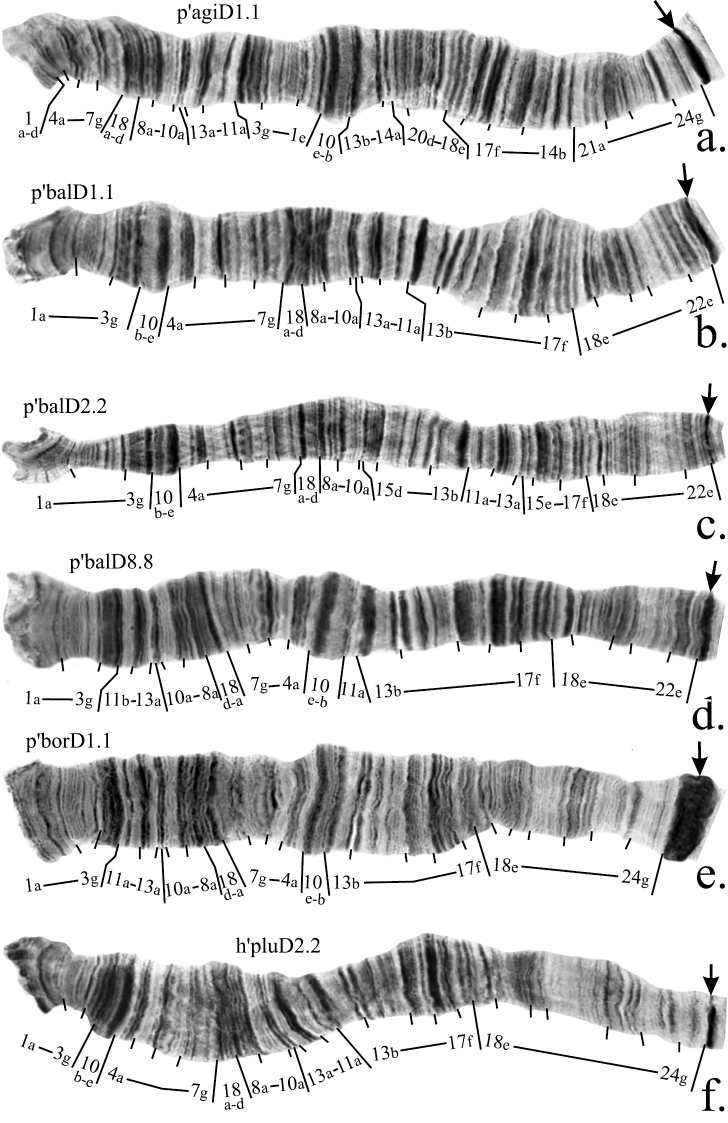
Mapping of banding sequences of *Chironomus plumosus* sibling species in arm D. **a** p’agiD1.1 **b** p’balD1.1 **c** p’balD2.2 **d** p’balD8.8 **e** p’borD1.1 **f** h’pluD2.2. Centromeric bands designated by arrows.

**Table 4.** Mapping of arm D main and alternative banding sequences in *Chironomus plumosus* group after the revision. † – main banding sequences are marked by *, ‡ – parts of the sequences highlighted in bold indicate regions which mapping had been changed as a result of the revision.

**Designation of banding sequence**	**Mapping of banding sequence**
p’agiD1*†	1a-d 4a-7g 18a-d 8a-10a 13a-11a 3g-1e 10e-b 13b**-14a 20d**-18e 17f-**14b 21a**-24g C ‡
p’agi2D1*	=p’agiD1
p’balD1	1a-3g 10b-e 4a-7g 18a-d 8a-10a 13a-11a 13b-17f 18e-22e C
p’balD2*	1a-3g 10b-e 4a-7g 18a-d 8a-**10a 15d-**13b 11a-13a **15e**-17f 18e-22e C
p’balD8	1a-3g **11b**-13a 10a-8a 18d-a 7g-4a 10e-b **11a** 13b-17f 18e-22e C
p’bonD1*	=p’pluD1
p’borD1*	=p’pluD1
h’borD2	=h’pluD2
h’entD1*	1a-**2c** 15e-16c 18d 8a-10a 13a-12a 18c-a 7g-4a 10e-b 13b-15d **2d**-3g 11a-c 16d-17f 18e-24g C
n’entD4	1a-**2c** 15e-16c 18d 8a-d 19h-18e 17f-16d 11c-a 3g-**2d** 15d-13b 10b-e 4a-7g 18a-c 12a-13a 10a-9a 20a-24g C
p’murD1	**1a-h** 11c-a 3g-**2d** 15d-13b 10b-e 4a-7g 18a-c 12a-13a 10a-8a 18d 16c-15e **2c-1i** 16d-17f 18e-24g C
p’murD2*	**1a-h 11c-a 3g-2d 15d-13b 15e-16c 18d 8a-10a 13a-12a 18c-a 7g-4a 10e-b 2c-1i 16d-17f 18e-24g C**
h’nudD1*	=h’entD1
p’nudD2	**1a-2c 15e-16c 18d 8a-d 20d-18e 17f-16d 11c-a 3g-2d 15d-13b 10b-e 4a-7g 18a-c 12a-13a 10a-9c 21ba 9ab 21c-24g C**
p’pluD1*	1a-3g 11a-13a 10a-8a 18d-a 7g-4a 10e-b 13b-17f 18e-24g C
h’pluD2	1a-3g 10b-e 4a-7g 18a-d 8a-10a 13a-11a 13b-17f 18e-24g C
p’sinD1*	1a-2g 13a 10a-8a 18d-a 7g-4a 10e-b 13b-14h 3g-2h 12d-11a 15a-17f 18e-24g C
h’spJD1*	=h’entD1
p’spKD1*	1a-3g 11a-13a 10a-8a 18d 16c-13b 10b-e 4a-7g 18a-c 16d-17f 18e-24g C
h’suwD1*	1a-3g 10b-e 4a-7g 18a-d 8a-10a 13a-11a 13b-17f 18e-24g C
p’useD1*	=p’pluD1
h’useD2	=h’pluD2

**1. Revision of arm D mapping of *Chironomus agilis* and *Chironomus* sp. prope *agili*s**

The banding sequences in arm D of both species are identical. They differ from p’pluC1 by two non-overlapping inversions. A correction should be made for breakpoints of the inversion in the proximal part of the arm: the left breakpoint falls between bands 14a and 14b instead of 14f and 14g, whereas the right breakpoint falls between regions 20 and 21 instead of bands 20b and 20c (Fig. 4a, Table 4).

**2. Revision of arm D mapping of *Chironomus balatonicus***

As was mentioned previously, *Chironomus balatonicus* differs from all other species of *Chironomus plumosus* group by the presence of a pericentric inversion inchromosome CD. Due to this, the arm D of *Chironomus balatonicus* is shorter than normal and consists of only 22 regions instead of 24.

*Chironomus balatonicus* has three banding sequences that could be found in homozygous state and, therefore, are considered in this study: p’balD1, p’ balD2 and p’balD8. Among them p’balD2 and p’balD8 require a minor revision. Banding sequence p’balD2 differ from p’balD1 by simple inversion, according to the previous mapping its right breakpoint was placed between regions 15 and 16, however it is clear that band 15e is not affected by the inversion so the real breakpoint falls between bands 15d and 15e (Fig. 4c, Table 4).

The banding sequence p’balD8 was considered previously as identical to p’pluD1 for all the arm length except the part affected by the pericentric inversion. However, our analysis has shown that this is not the case and p’pluD8 in fact originated from p’balD1 by a simple inversion. As a result, region 11 was broken into two parts and band 11a stayed between regions 10e-b and 13b-17f (Fig. 4d, Table 4).

**3. Revision of arm D mapping of *Chironomus entis*, *Chironomus muratensis*, *Chironomus nudiventris*, *Chironomus* sp. J, and *Chironomus* sp. K**

On the basis of our study we suggest that all banding sequences of these species have a common origin (Fig. 1d). We believe that the banding sequence p’spKD1 forms the basis of all other banding sequences of these species. It differs from p’pluD1 by a simple inversion (Fig. 4l, Table 4). The main banding patterns of *Chironomus entis*, *Chironomus nudiventris* and *Chironomus* sp. J are identical and originated from p’spKD1 by two inversion steps, correction was made for mapping of region 2: we believe that only bands 2a-c remain at the distal part of the arm whereas band 2d is affected by the inversion (Fig. 4k, Table 4). As n’entD4, p’nudD2, p’murD1 and p’murD2 are derivatives of h’entD1 and its homologous banding sequences, mapping of region 2 in them was also changed.

**Figure 4g–m.**
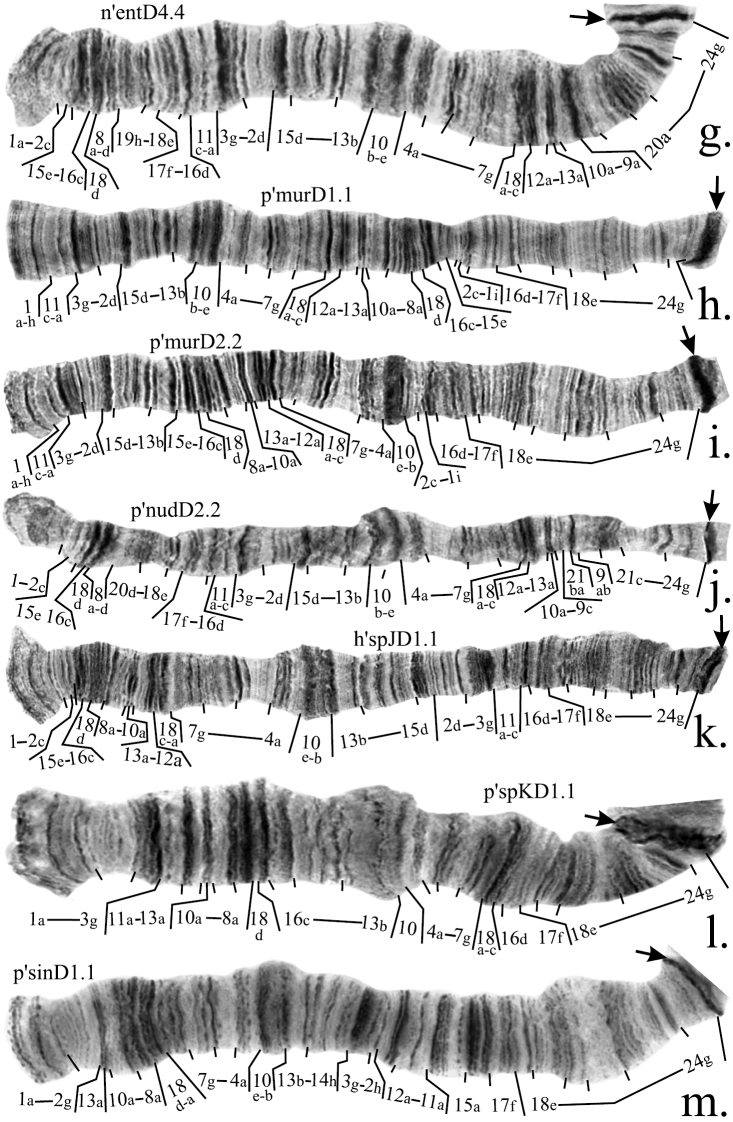
Mapping of banding sequences of *Chironomus plumosus* sibling species in arm D (*continued)*. **g** n’entD4.4 **h** p’murD1.1 **i** p’murD2.2 **j** p’nudD2.2 **k** h'spJD1.1 **l** p’spKD1.1 **m** p’sinD1.1.

Thorough analysis of p’nudD2 allowed us to conclude that it differs from h’nudD1, not by one, but by two inversions (Fig. 3e, 4j, Table 4). A small change was also made to the mapping of region 1 of p’murD1: according to previous mapping the left breakpoint was situated between regions 1 and 2, but our analysis had shown that the actual breakpoint falls between bands 1h and 1i (Fig. 4h, Table 4). Mapping of p’murD2 in the Keyl-Devai system is presented for the first time (Fig. 4i, Table 4).

Phylogenetic relationships of the revised banding sequences in arm D of the *Chironomus plumosus* group species are shown in Fig. 1d.

## Discussion

The revision of banding sequences in chromosome CD of *Chironomus plumosus* sibling species has shown that the phylogenetic relationships of banding sequences in both arms are more complex than appeared previously. The changes are not as significant as were made for arm A, for example (Golygina and Kiknadze 2008), but they still affect phylogenetic relationships of banding sequences of four species in arm C and six species in arm D (Fig. 1).

We have shown that banding sequences of *Chironomus entis*, *Chironomus muratensis*, *Chironomus nudiventris*, and *Chironomus* sp. J in arm C are more closely related than was considered previously and that p’entC2 of *Chironomus entis* can be considered as ancestral for banding sequences of other three species.

The most ancient banding sequence in arm C should be considered h’pluC2 and the identical banding sequences of *Chironomus agilis* and *Chironomus usenicus* as they are the closest to the p’pigC1.

In general, analysis of the phylogeny of banding sequences in arm C has shown that this arm has the highest level of divergence in comparison to arms A, B, and D, as only three clusters of homologous banding sequences exist in this arm, whereas there are four such clusters in arm A and D and seven in arm B (Fig. 1b, Golygina and Kiknadze 2008). Moreover, six species have species specific main and alternative banding sequences (Fig. 1b).

The revision in arm D mostly provided minor changes in the mapping of inversion breakpoints without affecting phylogenetic relationships of banding sequences in general. The only significant change has come from the correction of the inversion breakpoint of p’spKD1 which has made it the ancestor for all banding sequences of *Chironomus entis*, *Chironomus muratensis*, *Chironomus nudiventris*, *and Chironomus* sp J. In general, the banding sequences in arm D show a significant level of divergence with four species that have species specific main and alternative banding sequences and several complex inversions that distinguish banding sequences from one another.

Considering the high level of banding sequence divergence in both arms, it can be stated that chromosome CD is the most divergent among the three big chromosomes of *Chironomus* karyotype and probably plays a more important role in speciation than the other two.
